# A Study of the Records of One Hundred and Fifty-Five Cases of Operation for Appendicitis

**Published:** 1905-09

**Authors:** Charles A. Morton

**Affiliations:** Professor of Surgery in University College, Bristol; Surgeon to the Bristol General Hospital, and the Bristol Royal Hospital for Sick Children and Women


### A STUDY OF THE RECORDS OF ONE HUNDRED AND FIFTY-FIVE CASES OF OPERATION FOR APPENDICITIS.

BY

CHARLES A. MORTON, F.R.C.S.,

*Professor of Surgery in University College, Bristol; Surgeon to the Bristol General Hospital, and the Bristol Royal Hospital for Sick Children and Women.*

---

FIVE years ago I published some observations on 40 cases of operation for appendicitis.[1] Since then I have operated on 115 more (up to end of July of this year), and I think some further observations on the whole series may now be of interest. A table of the cases has been prepared. I now simply give the number of cases in the various forms of the disease in the second series.

Appendix (inflamed, ulcerated, perforated or gangrenous) removed during acute attack, no suppuration around, 8 cases, all recovered.

Localised suppuration around the appendix, 62 cases, 8 deaths.

Operation in quiescent period for recurrent attacks, 15 cases; after one severe attack, 1 case; chronic appendicitis, 1 case—all recovered.

Diffuse peritonitis, 14 cases, 12 recoveries.

General peritonitis, 6 cases, 1 recovery.

Mechanical obstruction associated with appendicitis, 4 cases, all died.

One, possibly two, cases of portal pyæmia. The undoubted case died. The other is included in the cases of removal of the appendix without suppuration, and recovered.

It may be well to define at once the difference between the cases I have labelled general peritonitis and those classified as

[1] *Bristol M.-Chir. J.*, 1900, xviii. 320.

diffuse peritonitis. By general peritonitis I mean cases in which the whole peritoneal cavity was involved; by diffuse peritonitis I mean a peritonitis spreading widely in the lower abdomen, and not merely suppuration localised just around the appendix. Such cases are frequently called general peritonitis.

In my first 40 cases there were 6 cases of general peritonitis, and all died. In my second series of 115 cases there were also 6 cases, with 1 recovery. There were, therefore, nearly two-thirds more cases in my first series. Is this due to a growing recognition of the need for earlier operation in severe cases? Nearly all the cases of diffuse peritonitis would undoubtedly have become fatal cases of general peritonitis if not operated on when they were. This series is particularly encouraging, as out of 14 cases 12 recovered. In all the cases the peritonitis was widespread; usually involving the pelvis, and often the whole of the lower abdomen, and without any localisation of the pus, or if there had been localisation, widespread infection had also occurred. Yet there was no evidence that the peritonitis extended all over the abdomen. In most I made a separate incision above the umbilicus to see if there was peritonitis in the upper abdomen, and did not find evidence of it. As I have already stated, many surgeons publish such cases as cases of recovery from general peritonitis, but general peritonitis is a much less hopeful condition to deal with, and the publication of such cases under the name of general peritonitis is misleading. All the cases of diffuse peritonitis were treated by free sponging and drainage, and the appendix was removed.

In my first series of cases there were many in which I evacuated the pus without opening the general peritoneal cavity, but very few in my second. This is because the cases came under my care at an earlier period, due also to an increasing recognition by medical men of the need for earlier operation.

I need not again refer to the method of evacuating the pus in such cases, as I did so fully in my last paper, but I now adopt an additional safeguard to prevent pus entering the general peritoneal cavity, which I did not do before. I never now operate on an acute appendicitis without placing the patient in such a sloping position that his right anterior superior spine is the lowest point of the trunk. There is no turning of the patient about when pus is found, he is already lying so that it will tend to run out of his wound. Of course very thorough gauze packing around the appendix region within the abdomen is also made before any adhesions are broken down.

In my second series are 8 cases (not including the case of portal pyæmia) in which, although the appendix was diseased, no pus was found around it and no spreading peritonitis. They all recovered. In one of these the appendix was actually gangrenous, in two perforated, and in another full of pus and greatly inflamed. These cases were saved considerable risk. In two there was acute inflammation with lymph on the surface, in one ulceration was associated with a temperature of 107°, and in another with dense old adhesions from previous attacks, and adhesions of coils of small intestines which were so bound down in a kinked condition as to render the patient liable to obstruction at any time.

In a few of the cases classified as localised abscess there were no adhesions: the pus lay around the appendix, and was kept from dissemination, apparently, simply by the pressure of surrounding distended coils. The danger of such a condition can be readily imagined, and the value of the position of the patient during operation, to which I have called attention, in such cases is evident.

There is no need to inquire as to the cause of death in the cases of peritonitis or mechanical obstruction, but it will be very useful to study the condition under which the localised collection of pus proved fatal, after operation, and I give an abstract of each case.

Case 1.—Boy, aged 12. Operation on third day; large collection of pus evacuated; no bad symptoms until three days later; then belly kept soft and free from distention and tenderness; there was no vomiting, but jaundice came on, and rales at bases of both lungs, with bronchial breathing at right base, and he died twenty-four hours later.

Case 2.—Male, aged 19. Not sent into hospital until eighth day; abscess opened at once; went on fairly well until forty-eight hours after; then symptoms of general peritonitis set in, and he died quickly. At the *post-mortem* the abscess was found communicating with peritoneal cavity as well as draining externally; it seemed well shut off at time of operation; probably adhesions had given way forty-eight hours after operation, but why?

CASE 3.—Boy, aged 7. Appendicular abscess on the left side; drained and appendix removed; operation 7 p.m.; condition good at end; rather restless, but otherwise all right when seen by house-surgeon 11.30 p.m.; hypodermic injection of morphia given later; not seen again by anyone but nurse, and died at 8 a.m.; examination of peritoneal cavity after death showed no spread of peritonitis from original focus.

CASE 4.—Boy, aged 5. Not sent into hospital until one week after onset; large abscess resonant with gas of decomposition; drained; next day rapid pulse, vomiting, and tender abdomen; general peritonitis diagnosed but none found *post-mortem*. Sloughs of the iliac muscle in floor of abscess sac; ? toxæmia. Highest temperature after operation 101°.

CASE 5.—Male, aged 36. Very large extra-peritoneal abscess, which I could not diagnose before operation, as it presented as an intense induration in the loin; drained; died five months later, after much hectic fever.

CASE 6.—Male, aged 37. Localised abscess drained and appendix removed; next day abdominal condition quite satisfactory, but cyanotic with tracheal rales audible when standing near him. R. 40, p. 120. Very little cough and no expectoration; breathing got worse in the night and he died. *Post-mortem* showed intense bronchitis, with collapse of the bases of lungs, but no spread of original focus of inflammation in the abdomen. Why did he get such bronchitis? He had had it shortly before operation, but had quite recovered. He only had chloroform, and not ether in any form. I believe a little vomit must have entered the air passages during the anæsthesia.

CASE 7.—Girl, aged 14. Large abscess outside ascending colon, with diverticula towards loin; two separate abscesses opened up during the removal of appendix. Was in satisfactory condition at end of operation, and remained so next day; no vomiting after operation, and abdomen less distended than before, soft, and not tender. Great restlessness and a very rapid pulse (without a temperature above 100°) set in at 2 a.m. second night, and she died following evening, without further symptoms. *Post-mortem* showed no extension of infection within the abdomen.

CASE 8.—Woman, aged 30. Abscess found ruptured on opening the abdomen a few hours after I first saw her; pus sponged out of pelvis and right loin; went on satisfactorily until third day after operation and then became jaundiced, and next day comatose, and died on fifth day with temperature of 105°. *Post-mortem* showed some lymph on a few pelvic coils. This was starting at time of operation, and had increased afterwards, but was certainly not enough to cause death. There was no suppuration in the liver. A general *post-mortem* was not allowed.

In none of these cases was the operation for the evacuation of the pus the cause of death. In case 2 it seemed possible that at the time the abscess was opened it also ruptured into the peritoneal cavity, but it appeared well shut off after the evacuation of the pus, and the symptoms of peritonitis did not arise until forty-eight hours later. In some of the cases there seemed to be a condition of toxæmia without high temperature which proved fatal, and the close resemblance of the symptoms to those of general peritonitis in case 4 is very instructive. In case 8 the jaundice was very intense, and it seems probable it was a case of acute yellow atrophy of the liver complicating appendicitis. In six out of the eight cases if the appendix had been removed directly the diagnosis of appendicitis could have been made, and life would probably have been saved.

In severe cases of appendicitis there may often be marked constipation, but there were three cases in which there was complete mechanical obstruction, from adhesions and kinking of the bowel in the wall of the abscess sac, and one of obstruction from old adhesions complicating a recent suppurative appendicitis. In two the appendix abscess was first opened, and then further operation was undertaken for the obstruction; in another the condition was obviously intestinal obstruction at the first operation, and the bowel was drained, as well as two abscess sacs; in the fourth the obstruction was due to old adhesions, but complicating recent suppurative appendicitis, and the symptoms of obstruction, did not come on until some days after the abscess was drained. All the cases were fatal. They were very obscure in nature from the combination of the signs of appendicitis and mechanical obstruction, and it may be of interest to give brief abstracts of them.

Case 1.—Boy, aged 12. History of origin that of appendicitis, but pain not first general and then in appendix region, but first in appendix region and then general. On admission to hospital very great general distension without general tenderness or rigidity, but tenderness in appendix region and behind bladder per rectum. Abscess evacuated and appendix removed. Large quantity of clear serous fluid in peritoneum and great distention of coils. That there was a true mechanical obstruction was not recognised at the operation, but as distension persisted and vomiting recurred abdomen again opened, and greatly distended coils traced to wall of abscess sac, where a coil was adherent and kinked, and below quite empty. Although coil separated and distended coils evacuated, paralytic obstruction persisted and the boy died.

CASE 2 was of the same nature, but the patient was a woman of 55. There was great tympanitic distension at the time the abscess was opened; this persisted, and a similar condition to that present in case 1 was found on a second operation.

CASE 3.—It was difficult from the symptoms to decide whether there was general peritonitis or mechanical obstruction. Usually cases of general peritonitis are mistaken for intestinal obstruction. In this case two large abscesses, from perforation of the appendix, were associated with obstruction from matting of the coils.

CASE 4.—In this case the obstruction was not present when the appendicitis abscess was opened, but came on some days later, and was found due to old adhesions in the pelvis from previous attacks of appendicitis.

In my first series of cases I had one of recovery from draining an abscess in the liver secondary to appendicular abscess previously operated on.[1] Since then I have had one fatal case of portal pyæmia, and one case of recovery after rigors and very high temperature, associated with very slight appendicitis, and as they are both of interest, I will give a brief abstract of them.

CASE 1.—Boy, aged 15. Taken ill four days before I saw him with abdominal pain, which was never severe; a temperature of 100°; no vomiting; on the following two days had several rigors, and temperature reached 105° more than once. Slight jaundice was first noticed on the fourth day. There was no swelling, and very little tenderness, in the appendix region or per rectum, but there was slight enlargement of, and tenderness over, the liver. I diagnosed portal pyæmia, but thought it would give him a chance if I removed the appendix, and did so next day. There was no pus around it, but a large patch of gangrene in its wall, and stinking pus in its interior. He had one rigor as he came out of the anæsthetic, but no more, though there was some pyrexia. The jaundice increased, the tenderness over the liver persisted; sometimes there was great distention of the stomach, with vomiting, relieved by passage of stomach tube, sometimes of the intestines, also easily removed by hot turpen-

[1] This case is fully reported in *Bristol M.-Chir. J.*, 1897, xv. 323.

tine enemata. The wound suppurated. He died ten days after the operation. No *post-mortem* could be made.

CASE 2.—In this case a mild and subsiding attack of appendicitis was followed by severe rigors, but on removal of the appendix a rapid recovery was made. I do not think we can regard the diagnosis of portal pyæmia as certain, but it is difficult otherwise to account for the symptoms. We must remember the way in which cases of aural pyæmia recover when the primary focus of septic phlebitis is dealt with. In this case such a primary focus may have existed in a vein in the ulcerated appendix.

Abdominal pain and vomiting came on four days before I saw him, and the pain persisted in the appendix region, but he continued to travel about the country until the end of the fourth day, when he returned home. Then he sent for his doctor, who found the temperature was 102°, but the pain was much less. In the night the temperature reached 105°, with a rigor, and at 10 a.m. was found by his doctor to be 108°, and he had another severe rigor. The temperature was taken by his doctor, who checked the registrations of the thermometer used. I saw him that afternoon; there was no tenderness over the liver and no jaundice, but a small tender swelling in the appendix region. I removed the appendix that evening. It was very adherent, and the mucous membrane was ulcerated. There were no further rigors, and no tenderness developed over the liver, but distinct jaundice was present on the day after operation.

In connection with these cases I may call attention to the very high temperature with a marked rigor sometimes present at the onset of appendicitis. I had one case in which the temperature rose to 107° with a severe rigor within 24 hours of the onset of pain. The appendix was gangrenous.

I cannot give space in this paper to refer to the cases of peritonitis, or those of operation in the quiescent period for recurrence, in detail; but I would repeat what I have elsewhere stated, that it is not possible to separate cases of well localised acute appendicitis from those in which the peritonitis is not localised, but spreading. In the latter cases the pulse may not reach 100; the patient may not appear very ill, and the symptoms may not seem particularly urgent, and yet when we open the abdomen we find pus and lymph all over the pelvis, and perhaps the right loin as well. I have published in detail one or two such cases,[1] and I remember another in which the

[1] *Lancet*, 1901, i. 1134; *Brit. M. J.*, 1902, i. 325.

house-surgeon told me that when I saw the patient I should decide not to operate, but fortunately I did so.

There are also many questions which I should like to try and answer from a study of the records of these cases, such as the relationship between the symptoms and the condition of the appendix found at the operation, and the state of the appendix leading to such attacks, but neither have I time at present, nor would there be space in the Journal for so long a paper. That appendicitis is not always a peritonitis around the appendix, as one surgeon has stated, I am quite certain, for I have records of cases in which the symptoms of acute appendicitis were present, but the appendix was already buried in adhesions. That such adhesions are often present without any history (carefully inquired for) of previous attacks of appendicitis I am equally sure, and hence it is evident that a dry adhesive peritonitis may be present around the appendix, either without any symptoms or such as to be forgotten by the patient. That the pain of appendicitis is usually epigastric, or umbilical, or general, at the commencement, and not in the appendix region until later, is not yet fully recognised by all, and hence acute indigestion or colic is the label often put on the case at first; and possibly in many cases no other diagnosis may be possible, but a raised temperature might be found in most, if looked for. Of referred pains, I have known the rectum, the anus, and the testis as the seat. The presence of referred pain in the latter in appendicitis adds to the difficulty in the diagnosis of some cases from renal colic. In a few cases I have found that vomiting and diarrhœa preceded the pain, but this is very unusual, and until lately I should have doubted if they ever did so in appendicitis.

(*To be continued.*)

